# Liver Antioxidant Capacity and Steatosis in Laying Hens Exposed to Various Quantities of Lupin (*Lupinus angustifolius*) Seeds in the Diet

**DOI:** 10.3390/antiox13020251

**Published:** 2024-02-19

**Authors:** Marta Wójcik, Sebastian Grabowski, Łukasz S. Jarosz, Bartłomiej Szymczak, Vincenzo Longo, Clara Maria della Croce, Marcin Hejdysz, Adam Cieślak, Kamil Gruszczyński, Agnieszka Marek

**Affiliations:** 1 Sub-Department of Pathophysiology, Department of Preclinical of Veterinary Sciences, Faculty of Veterinary Medicine, University of Life Sciences in Lublin, Akademicka 12, 20-033 Lublin, Poland; marta.wojcik@up.lublin.pl (M.W.); bartlomiej.szymczak@up.lublin.pl (B.S.); kamil.gruszczynski@animal-pharma.com (K.G.); 2 Department of Epizootiology and Clinic of Infectious Diseases, Faculty of Veterinary Medicine, University of Life Sciences in Lublin, Głęboka 30, 20-612 Lublin, Poland; sebastian.grabowski@vp.pl; 3 Institute of Agricultural Biology and Biotechnology, National Research Council (CNR), Via Moruzzi 1, 56124 Pisa, Italy; longo@ibba.cnr.it (V.L.); dellacroce@ibba.cnr.it (C.M.d.C.); 4 Department of Animal Breeding and Product Quality Assessment, Poznań University of Life Sciences, Wołynska 33, 60-637 Poznań, Poland; marcin.hejdysz@up.poznan.pl; 5 Department of Animal Nutrition and Feed Management, Poznań University of Life Sciences, Wołyńska 33, 60-637 Poznań, Poland; adam.cieslak@up.poznan.pl; 6 Department of Preventive Veterinary and Avian Diseases, Faculty of Veterinary Medicine, University of Life Sciences in Lublin, 20-950 Lublin, Poland; agnieszka.marek@up.lublin.pl

**Keywords:** hens, *Lupinus angustifolius*, lipid peroxidation, antioxidant capacity, antioxidant enzymes, DT-diaphorase, ethoxycoumarin O-deethylase

## Abstract

Despite the many beneficial properties of legume plants, their use in diets for poultry is limited by the presence of antinutritional factors. The aim of the study was to determine the activity of DT-diaphorase, ethoxycoumarin O-deethylase, and catalase, and the concentration of malondialdehyde in liver tissue, as well as the activity of SOD and CAT in the serum of Hy-line Brown hens fed a diet supplemented with various doses of *Lupinus angustifolius* seeds. The results indicate that the use of large amounts of lupin in the diet resulted in an increase in MDA concentration in the liver and the lipid vacuolization of hepatocytes. A significant increase in DTD activity was observed in chickens receiving 15% lupin. Regardless of lupin dose, no increase in SOD activity was observed in chicken serum after 33 days of the experiment. From the 66th day of the experiment, an increase in catalase activity in the serum of laying hens was observed, while low activity of this enzyme was found in the liver. It can be concluded that the short-term use of lupin in the diet of laying hens does not affect the activity of antioxidant enzymes and, therefore, does not affect the oxidative–antioxidant balance of their body.

## 1. Introduction

The breeding of laying hens, which are used to produce table eggs and chicks, is currently one of the fastest growing sectors of poultry production [1]. Production indicators obtained in this sector, including laying rates and egg quality, depend mainly on diet [2]. Chickens have high feed requirements in comparison with other animal species [3,4]. Therefore, to obtain high production indicators, feed rations must be properly balanced for energy and the content of protein, amino acids, and minerals [5]. One of the most commonly used protein sources in diets for laying hens is soybean extraction meal, which contains large amounts of protein of high biological value [6]. Most of the soybean meal used in poultry diets is produced from the seeds of genetically modified plants, which raises justified concerns among consumers regarding the health of products obtained from laying hens [7,8,9]. These conditions encourage the search for alternative, high-protein feed ingredients for poultry, comparable to soybean economically and in terms of quality [9]. Currently, legume plants are often used as a substitute source of plant protein in diets for laying hens [10]. These include lupin, especially narrowleaf lupin (*Lupinus angustifolius*) [8,11].

Due to the presence of numerous bioactive substances, including proteins, peptides, alkaloids, and phenolic compounds, lupin exhibits hypocholesterolemic, antioxidant, antimicrobial, and anti-inflammatory activity [12,13]. The health-promoting effects of lupin have been tested in clinical trials in humans and in animal models. Particularly noteworthy is the anti-inflammatory effect of proteins contained in lupin, which inhibit the action of enzymes involved in the inflammatory pathway, such as phospholipase A2 (PLA2), cyclooxygenase 2 (COX-2), thrombin, and transglutaminase (TG), and reduce the proliferation of mononuclear cells in peripheral blood (PBMC) and the level of pro-inflammatory cytokines of T helper lymphocytes (Th1, Th9 and Th17) [14]. The proteins contained in lupin also have hypocholesterolemic effects by reducing the levels of triglycerides, total cholesterol, and low-density lipoproteins (LDLs), and by reducing the expression of SREBP-1c and HMG-CoA reductase genes they also guarantee a hepatoprotective effect [15,16,17]. Owing to these properties, the use of lupin as a supplement in the diet reduces the risk of certain types of cancer [10], degenerative diseases, cardiovascular disease, hypertension, type 2 diabetes, and obesity in people [18,19]. Despite the many beneficial properties of lupin, its use in diets for poultry is limited by the presence of antinutritional factors, such as alkaloids, saponins, lectins, phytates, trypsin, chymotrypsin inhibitors, and tannins, which negatively affect weight gain and the health of poultry [12,20].

Toxic quinolizidine alkaloids contained in lupin have adverse effects on the human body, leading to damage to the nervous, reproductive, and digestive systems [21]. Research on rats [22] has also shown that the use of *Lupinus angustifolius* in the diet in amounts of 25.8, 26.6, and 27.1% of basal feed leads to congestion of the intralobular capillaries and parenchymatous and the vacuolar degeneration of hepatocytes due to the effects of toxic alkaloids on liver cells, ultimately resulting in the degeneration of the organ. Contrasting results were obtained in a study in mice [23], which showed that bioactive proteins isolated from *Lupinus angustifolius*, including the protein hydrolysate GPETAFLR, exhibit anti-inflammatory properties, reducing the mRNA expression of pro-inflammatory cytokines TNF-α and IL-6 and increasing the expression of anti-inflammatory IL-10 in the liver. This results in a reduction in the concentrations of markers of liver damage, AST, ALT, ALP, and LDH, and in the accumulation of fat in the liver, which affects the signaling pathways involved in the regulation of gene expression and the activity of proteins involved in lipid metabolism [23]. These authors also demonstrated that GPETAFLR inhibits the development of non-alcoholic fatty liver disease (NAFLD) in mice [23]. It is worth noting that despite the increasingly common use of lupin in diets for laying hens, no in-depth studies have investigated its effect on the structure of the internal organs responsible for metabolic processes, e.g., the liver. The low fat content in lupin might suggest that its long-term use would have positive effects in chickens, especially with regard to liver steatosis. Therefore, one of the purposes of this study was to learn the actual effect of long-term supplementation with varying amounts of lupin on liver steatosis, and thus the impairment of the metabolic functions of the liver, especially antioxidant processes.

Apart from toxic alkaloids, lupin also contains numerous phytochemicals, including polyphenols, phytosterols, and squalene [24,25]. In addition, lupin contains carotenoids, such as β-carotene and zeaxanthin [26,27,28]. The presence of phenolic compounds and carotenoids determines one of the most important properties of lupin, i.e., its antioxidant activity, owing to which its use as a feed supplement protects against oxidative stress and increases the body’s ability to eliminate free radicals responsible for oxidation processes in cells [29]. Antioxidant activity has also been demonstrated for certain peptide fractions obtained from *L. angustifolius* protein that can be used to produce antioxidant nutraceuticals for use in humans [30]. The antioxidant properties of diet supplements, in addition to their significance in the human diet, are also important in laying hens, which provide table eggs [31]. This group of farm animals is susceptible to the effects of environmental, technological, and dietary stressors, resulting in the development of oxidative stress. Excessive ROS production under conditions of oxidative stress causes lipid peroxidation, mainly of the polyunsaturated fatty acids (PUFAs) present in the cell membrane, leading to cell membrane damage and the oxidation of proteins and DNA [32]. These phenomena induce damage to parenchymal organs, such as the liver, leading to liver failure [32,33]. A beneficial antioxidant effect can be expected after using lupin as a feed additive for laying hens, but its use is limited by the presence of antinutritional factors. Currently, in accordance with poultry feeding standards [34], the amount of lupin in feed should not exceed 15% of the diet. Studies by Van Barneveld [35] and Perez-Maldonado et al. [36], on the other hand, have shown that the inclusion of lupin in diets for laying hens in the amount of 10–20%, or even 25%, has no negative effect on production, provided the diet is additionally supplemented with amino acids.

Despite numerous studies on the effects of narrowleaf lupin (*Lupinus angustifolius*) used as a feed additive on production parameters, the amount of lupin that would be completely safe for poultry and guarantee strong antioxidant properties protecting cells against damage has not been established. The literature also contains no information on the effects of the long-term use of lupin as a feed additive on processes of liver degeneration and steatosis in laying hens.

Therefore, we hypothesized that the long-term inclusion of high levels of lupin (20–25%) in feed for laying hens increases lipid peroxidation and limits antioxidant activity in the birds, which leads to cell damage and degenerative processes in the parenchymal organs, e.g., the liver. To verify this hypothesis, we determined the activity of NADPH quinone reductase (DT-diaphorase), ethoxycoumarin O-deethylase (ECOD), and catalase (CAT), and the concentration of malondialdehyde (MDA) in the liver tissue, as well as SOD and CAT activity in the serum of laying hens fed a diet with lupin seeds (*Lupinus angustifolius*) in amounts of 0%, 10%, 15%, 20, or 25%. A microscopic evaluation of HE-stained liver specimens was performed as well.

## 2. Materials and Methods

### 2.1. Ethical Statement

The experiment was carried out at the Experimental Station of the Poznań University of Life Sciences, Gorzyń 4, Międzychód commune. The study was conducted according to the guidelines of the Local Ethics Committee for Animal Testing at the University of Life Sciences in Lublin, Poland, with respect to animal experimentation and care of the animals under study (Resolution No. 86/2023 titled “The influence of a feed mixture enriched with lupin grains on feed digestibility, health and production parameters, and indicators of the humoral and cellular immune response of laying hens”).

### 2.2. Animals and Diets

In total, 300 Hy-line Brown hens at the age of 17 weeks were weighed and randomly placed in individual metal cages (84 × 46 cm) with a metal wire floor (3 birds in each cage) with ad libitum access to drinking water and feed. Animals were fed pre-laying diets based on wheat, maize, and soybean meal and containing 16% crude protein and 11 MJ kg^−1^ ME/kg from 17 to 24 weeks of age. Before the laying period, the birds were randomly assigned to five treatments, each with 60 hens (20 replicates in each/3 hens per replicate). The experiment began at 24 weeks of age and was completed at 39 weeks of age. The lighting program was 14 h of light and 10 h of darkness.

Birds from the control and experimental groups were fed a diet based on wheat/native grain and soybean meal as the main protein sources. The diets were formulated according to nutritional recommendations by the National Research Council [37] and the Hy-Line Brown Commercial Management Guide 2002–2004 [38]. All hens were fed the same basal diet during the experiment (Table 1). The feed given to laying hens was in the form of a mash. No antibiotic growth promoters or antibiotic treatments were used during the entire experimental period.

Birds from the control group (T0) were fed a basal diet without lupin seeds. For the hens in groups T10–T25, the experimental groups, raw lupin seeds (*Lupinus angustifolius*) were added to the basal diet in amounts of 10%, 15%, 20%, and 25%, respectively (Table 1). The nutrient content of the diets was calculated on the basis of the chemical composition of the raw feedstuffs and metabolizable energy value (Table 2). Throughout the experimental period, the hens had free access to drinking water and feed. The lighting program was 14 h of light and 10 h of darkness.

### 2.3. Blood and Tissue Samples

The material for analysis comprised 2 mL samples of peripheral blood from the wing vein. The samples were collected in sterile vacuum tubes containing a clot activator and a serum separator (Vacuette, Medlab Products, Raszyn, Poland) at 29 weeks of age (33rd day of experiment), 34 weeks of age (66th day of experiment), and 39 weeks of age (99th day of the experiment). At each stage of the experiment, 20 blood samples were collected from the control group and each experimental group (1 hen from each replicate/20 hens per group). The samples were transported to the laboratory at +4 °C to +8 °C within 1 h. Serum was obtained by centrifuging the blood at room temperature (20–22 °C) for 15 min at 4000× *g*. The serum was apportioned and stored at −80 °C until analysis.

Moreover, blood was collected from 20 randomly selected hens at 24 weeks of age before the start of the experiment (group T00), in order to determine the activity of the antioxidant enzymes catalase (CAT) and superoxide dismutase (SOD) in the serum.

Liver samples were collected from 20 birds in each group, from the same birds as the blood samples, and stored at −80 °C until further analysis.

### 2.4. Histopathological Examination of Liver Tissue

**Figure 1:**
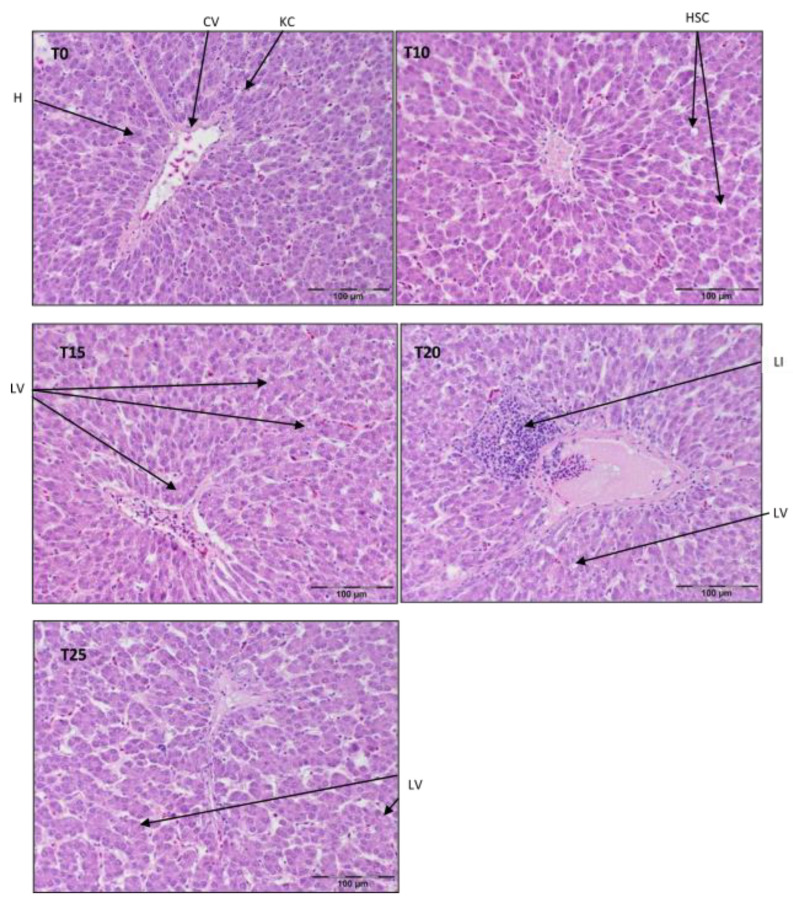
Light microscopic histological appearance of liver tissue obtained from each of group of birds at day 33 of experiment (29 weeks of age) (representative photos with the most clearly visible phenotyping histopathological changes, the same observed in other slides, derived from the same group of birds). H/E staining was used for histological observation. Original magnification ×200. The structure of the liver tissue was examined using graphic analysis software (OlympuscellSens Version 1.5; Olympus, Tokyo, Japan). H—hepatocytes; CV—central vein; HSC—hepatic stellate cells; KC—Kupfer cells; LI—lymphatic infiltration; LV—lipid vacuolization; T0—basal feed; T10—basal feed + 10% raw lupin seeds (*Lupinus angustifolius*); T15—basal feed + 15% raw lupin seeds (*Lupinus angustifolius*); T20—basal feed + 20% raw lupin seeds (*Lupinus angustifolius*); T25—basal feed + 25% raw lupin seeds (*Lupinus angustifolius*).

**Figure 2:**
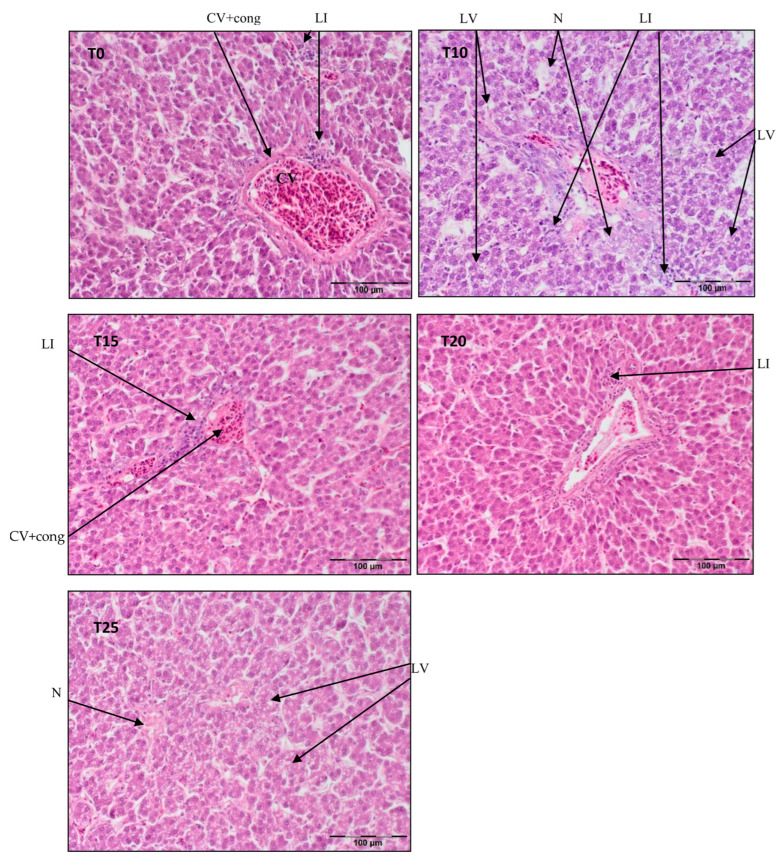
Light microscopic histological appearance of liver tissue obtained from each of group of birds at day 66 of experiment (34 weeks of age) (representative photos with the most clearly visible phenotyping histopathological changes, the same observed in other slides, derived from the same group of birds). H/E staining was used for histological observation. Original magnification ×200. The structure of the liver tissue was examined using graphic analysis software (OlympuscellSens Version 1.5; Olympus, Tokyo, Japan). CV+ cong—central vein with congestion; LI—lymphatic infiltration; LV—lipid vacuolization; N—necrosis; T0—basal feed; T10—basal feed + 10% raw lupin seeds (*Lupinus angustifolius*); T15—basal feed + 15% raw lupin seeds (*Lupinus angustifolius*); T20—basal feed + 20% raw lupin seeds (*Lupinus angustifolius*); T25—basal feed + 25% raw lupin seeds (*Lupinus angustifolius*).

**Figure 3:**
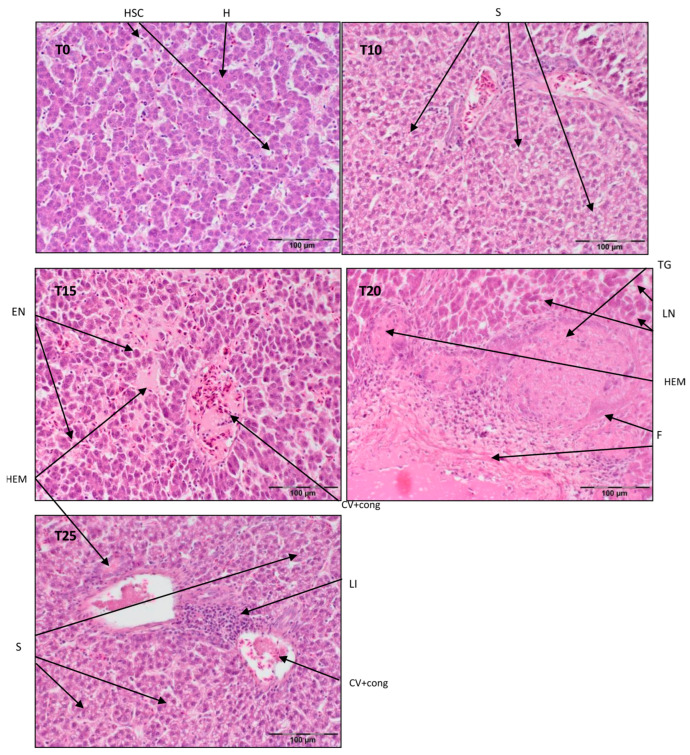
Light microscopic histological appearance of liver tissue obtained from each of group of birds at day 99 of experiment (39 weeks of age) (representative photos with the most clearly visible phenotyping histopathological changes, the same observed in other slides, derived from the same group of birds). H/E staining was used for histological observation. Original magnification ×200. The structure of the liver tissue was examined using graphic analysis software (OlympuscellSens Version 1.5; Olympus, Tokyo, Japan). H—hepatocytes; HSC—hepatic stellate cells; LI—lymphatic infiltration; EN—early necrosis; LN—late necrosis; F—fibrosis; S—steatosis; TG—tumorigenesis; CV+ cong—central vein with congestion; HEM—hemorrhage; T0—basal feed; T10—basal feed + 10% raw lupin seeds (*Lupinus angustifolius*); T15—basal feed + 15% raw lupin seeds (*Lupinus angustifolius*); T20—basal feed + 20% raw lupin seeds (*Lupinus angustifolius*); T25—basal feed + 25% raw lupin seeds (*Lupinus angustifolius*).

A portion of each liver tissue sample (5 groups × 20 birds × 3 slides) taken from the right lobe (≈1 cm^2^) was fixed for 24 h at room temperature in 10% phosphate-buffered formalin (pH 7.0), dehydrated and cleared with Ottix Plus and Ottix Shaper solvent substitutes (DiaPath, Martinengo, Italy), and then embedded in paraffin in a tissue processor (STP 120, Waltham, MA, USA). Further, 4 μm thick tissue samples were cut with a microtome (Microm HM 360, Microm, Walldorf, Germany) and stained in standard hematoxylin–eosin (H/E 5:5 min.) protocol for histology. Stained sections were observed in normal light with a light microscope (Olympus BX63) equipped with a camera (Olympus XC50, Olympus, Tokyo, Japan) at magnification ×200 [39]. Representative photos of histological preparations were selected for the study, in which clear structural changes were visible and occurred in other samples from the same time point and group of birds. See Figure 1, Figure 2 and Figure 3.

### 2.5. Determination of Malondialdehyde (MDA) Concentrations in Tissue

The MDA concentration in tissue homogenates was assessed using a colorimetric method, with absorbance measured in the butanol layer containing the TBA-MDA complex (TBA—thiobarbituric acid) at a wavelength of 552 nm, as described in Wójcik et al. [40]. The MDA concentration was determined using a standard curve obtained using malondialdehyde bis-dimethylacetal. The Lowry method was used to determine the protein levels in the samples according to Maehre et al. [41].

### 2.6. Isolation of Microsomal and Cytosolic Fractions

Microsomal and cytosolic fractions were obtained by two-step ultracentrifugation as described previously [42]. The liver homogenate in a phosphate buffer (100 mM K_2_HPO_4_—dipotassium phosphate, 1.15% KCl—potassium chloride, 1 mM EDTA—ethylenediaminetetraacetic acid, pH 7.4) was centrifuged for 30 min at 9000× *g*, and the supernatant was collected and transferred to ultracentrifugation tubes.

In the first step of ultracentrifugation (170 min at 100,000× *g*), the cytosolic fraction was obtained from the supernatant. The pellet was resuspended in the phosphate buffer and centrifuged under the same conditions. Lastly, the pellet containing the microsomal fraction was resuspended in a resuspending buffer (100 mM K_2_HPO_4_, 1.15% KCl, 0.1 mM EDTA, pH 7.4). Both the cytosolic and microsomal fractions were stored at −80 °C for further analysis.

### 2.7. Evaluation of DT-Diaphorase Activity

The activity of DT-diaphorase (NADPH quinone reductase) was measured in the cytosolic fraction [43]. This activity was determined by analyzing the reduction of DCPIP (dichlorophenolindophenol) by NADPH. Samples were incubated for 1 min, and absorbance was measured every 15 s for 2 min at 630 nm in a double-beam spectrophotometer (Lambda 365, PerkinElmer, Shelton, WN, USA). The results are expressed in nmol/1 mg protein/min.

### 2.8. Assessment of Ethoxycoumarin O-Deethylase Activity

Ethoxycoumarin O-deethylase (ECOD) was assessed in microsomal fraction by the quantification of umbelliferone production [44]. A mixture consisting of 1 mM ethoxycoumarin, 5 mM MgCl_2_ (magnesium chloride), an NADPH-generating system, and 0.25 mL of the sample was incubated for 5 min at 37 °C. The reaction was stopped by adding 0.85 mL of 0.31 M TCA (trichloroacetic acid). The pH of the mixture was adjusted to approximately 10 with 0.65 mL of 1.6 M NaOH–glycine buffer (pH 10.3). The quantity of umbelliferone was determined fluorimetrically (ISA Fluoromax II; HORIBA Scientific, Northampton, UK), with excitation at 390 nm and emission at 440 nm.

### 2.9. Evaluation of Catalase Activity

The enzymatic activity of catalase was spectrophotometrically determined in the cytosolic fraction at a wavelength of 240 nm in a double-beam spectrophotometer (Lambda 365, PerkinElmer). The samples were zeroed, and then 300 μL of phosphate buffer was added to one cuvette, while 300 μL of 30 mM H_2_O_2_ was added to the other. After one-minute incubation, a measurement was taken every 15 s for a period of 2 min. The results were corrected for protein content measured by the Lowry method, and enzymatic activity was expressed in nmol/1 mg protein/min [45].

### 2.10. Assay of Catalase and Superoxide Dismutase in Chicken Serum

ELISA kits (Cayman Chemical, Ann Arbor, MI, USA no. 707002 and 706002) were used to determine catalase (CAT) and superoxide dismutase (SOD) in the chicken serum. All assays were performed according to the producer’s instructions. All samples were tested in triplicate.

### 2.11. Statistical Analysis

**Figure 4:**
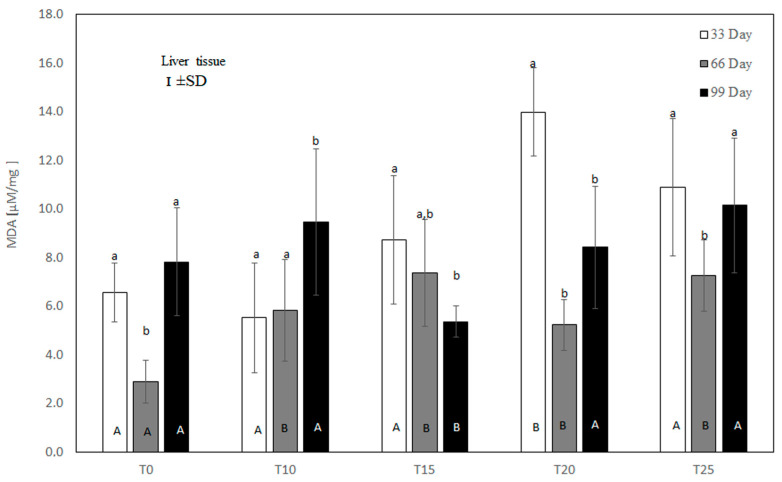
One-way ANOVA and Tukey tests (*p* < 0.05) for MDA concentration in the liver of hens at day 33 of experiment (29 weeks of age), day 66 of experiment (34 weeks of age), and day 99 of experiment (39 weeks of age). The same letter designations indicate no statistically significant differences. Upper-case letters were used to show statistically significant results (*p* ≤ 0.05) between groups, and lower-case letters indicate differences within the group on the three study dates. T0—basal feed; T10—basal feed + 10% raw lupin seeds (*Lupinus angustifolius*); T15—basal feed + 15% raw lupin seeds (*Lupinus angustifolius*); T20—basal feed + 20% raw lupin seeds (*Lupinus angustifolius*); T25—basal feed + 25% raw lupin seeds (*Lupinus angustifolius*). ±SD—standard deviation.

**Figure 5:**
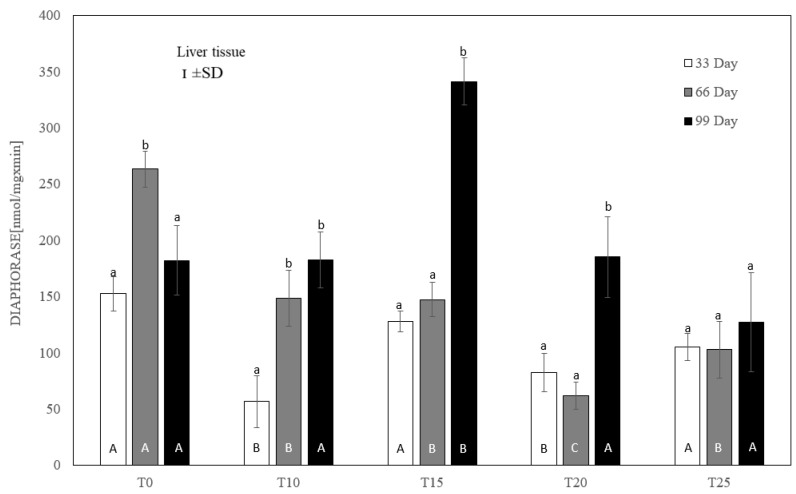
One-way ANOVA and Tukey tests (*p* < 0.05) for cytosolic *DT-diaphorase* activity in the liver of hens at day 33 of experiment (29 weeks of age), day 66 of experiment (34 weeks of age) and day 99 of experiment (39 weeks of age). The same letter designations indicate no statistically significant differences. Upper-case letters were used to show statistically significant results (*p* ≤ 0.05) between groups, and lower-case letters indicate differences within the group on the three study dates. T0—basal feed; T10—basal feed + 10% raw lupin seeds (*Lupinus angustifolius*); T15—basal feed + 15% raw lupin seeds (*Lupinus angustifolius*); T20—basal feed + 20% raw lupin seeds (*Lupinus angustifolius*); T25—basal feed + 25% raw lupin seeds (*Lupinus angustifolius*). ±SD—standard deviation.

**Figure 6:**
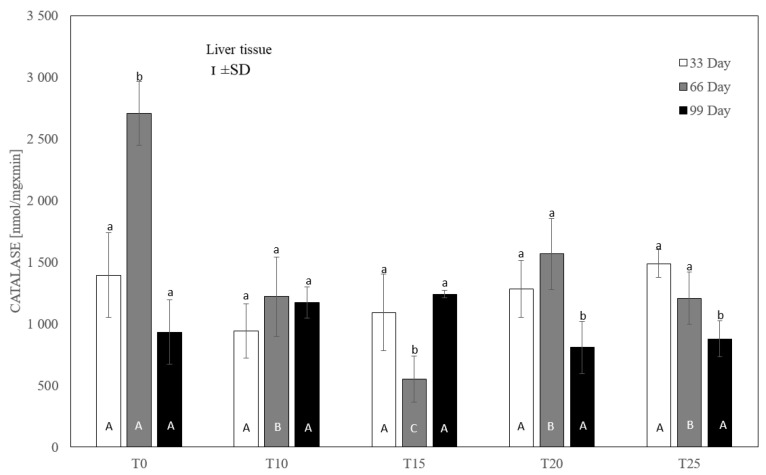
One-way ANOVA and Tukey tests (*p* < 0.05) for cytosolic catalase (CAT) activity in the liver of hens at day 33 of experiment (29 weeks of age), day 66 of experiment (34 weeks of age) and day 99 of experiment (39 weeks of age). The same letter designations indicate no statistically significant differences. Upper-case letters were used to show statistically significant results (*p* ≤ 0.05) between groups, and lower-case letters indicate differences within the group on the three study dates. T0—basal feed; T10—basal feed + 10% raw lupin seeds (*Lupinus angustifolius*); T15—basal feed + 15% raw lupin seeds (*Lupinus angustifolius*); T20—basal feed + 20% raw lupin seeds (*Lupinus angustifolius*); T25—basal feed + 25% raw lupin seeds (*Lupinus angustifolius*). ±SD—standard deviation.

**Figure 7:**
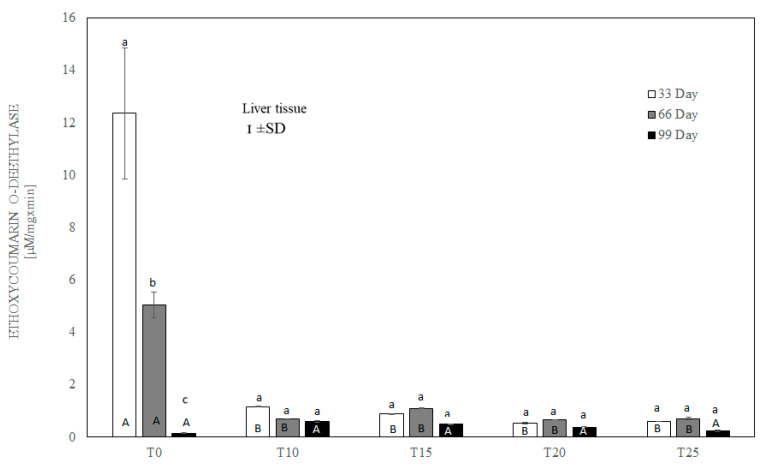
One-way ANOVA and Tukey tests (*p* < 0.05) for ethoxycoumarin O-deethylase (ECOD) activity in the liver of hens at day 33 of experiment (29 weeks of age), day 66 of experiment (34 weeks of age) and day 99 of experiment (39 weeks of age). The same letter designations indicate no statistically significant differences. Upper-case letters were used to show statistically significant results (*p* ≤ 0.05) between groups, and lower-case letters indicate differences within the group on the three study dates. T0—basal feed; T10—basal feed + 10% raw lupin seeds (*Lupinus angustifolius*); T15—basal feed + 15% raw lupin seeds (*Lupinus angustifolius*); T20—basal feed + 20% raw lupin seeds (*Lupinus angustifolius*); T25—basal feed + 25% raw lupin seeds (*Lupinus angustifolius*). ±SD—standard deviation.

**Figure 8:**
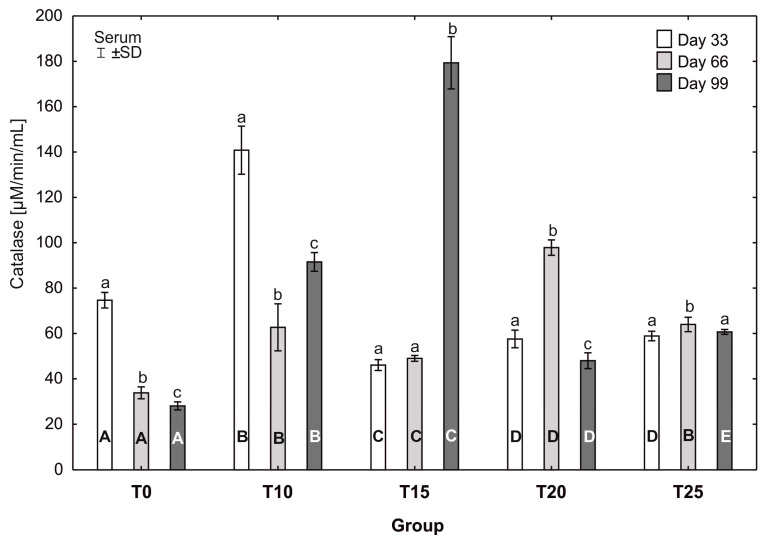
One-way ANOVA and Tukey tests (*p* < 0.05) for catalase (CAT) activity in the serum of hens at day 33 of experiment (29 weeks of age), day 66 of experiment (34 weeks of age), and day 99 of experiment (39 weeks of age). The same letter designations indicate no statistically significant differences. Upper-case letters were used to show statistically significant results (*p* ≤ 0.05) between groups, and lower-case letters indicate differences within the group on the three study dates. T0—basal feed; T10—basal feed + 10% raw lupin seeds (*Lupinus angustifolius*); T15—basal feed + 15% raw lupin seeds (*Lupinus angustifolius*); T20—basal feed + 20% raw lupin seeds (*Lupinus angustifolius*); T25—basal feed + 25% raw lupin seeds (*Lupinus angustifolius*). ±SD—standard deviation.

**Figure 9:**
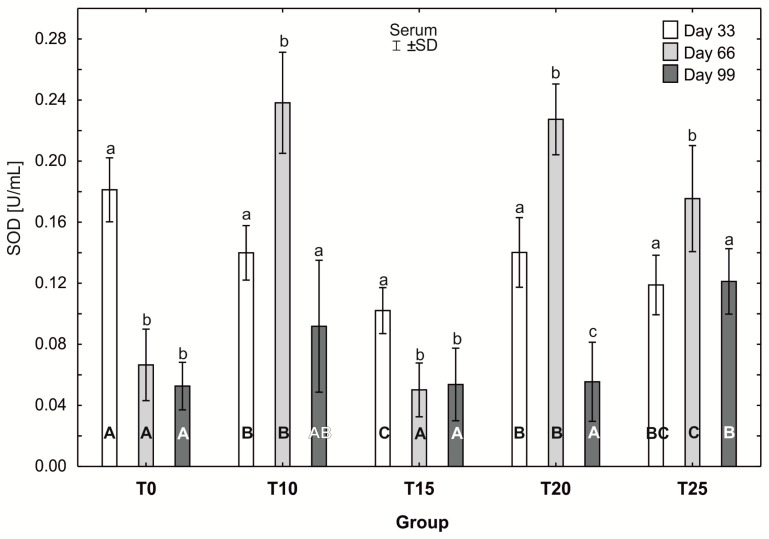
One-way ANOVA and Tukey tests (*p* < 0.05) for superoxide dismutase (SOD) activity in the serum of hens at day 33 of experiment (29 weeks of age), day 66 of experiment (34 weeks of age), and day 99 of experiment (39 weeks of age). The same letter designations indicate no statistically significant differences. Upper-case letters were used to show statistically significant results (*p* ≤ 0.05) between groups, and lower-case letters indicate differences within the group on the three study dates. T0—basal feed; T10—basal feed + 10% raw lupin seeds (*Lupinus angustifolius*); T15—basal feed + 15% raw lupin seeds (*Lupinus angustifolius*); T20—basal feed + 20% raw lupin seeds (*Lupinus angustifolius*); T25—basal feed + 25% raw lupin seeds (*Lupinus angustifolius*). ±SD—standard deviation.

After testing the data for normality (Shapiro–Wilk test) and homoscedasticity (Levene’s test), analysis of variance (ANOVA) was performed on different sets (groups T0-T25 in three terms) of data, followed by Tukey’s test (*p* ≤ 0.05). The normal distribution of the data and the homogeneity of the variances made it possible to carry out parametric statistical tests to check the statistical differences between the groups analyzed, namely one-factor analysis of variance with a Tukey post hoc test and a *t*-test. The results are expressed as mean and ±standard deviation (±SD), and differences were considered significant at *p* ≤ 0.05. Capital letters were used to indicate statistically significant differences between groups on the same day, and lower-case letters were used to indicate differences between groups in three terms. Statistical analyses were performed using Statistica 13.2 software (StatSoft, Inc., Krakow, Poland). See Figure 4, Figure 5, Figure 6, Figure 7, Figure 8 and Figure 9.

In turn, an independent *t*-test was used to determine whether there was a significant difference between the means of two groups: the T00 reference group with the other experimental groups, respectively (Table S1).

## 3. Results

### 3.1. Liver Histopathology

Referring to Figure 1, a typical structure of liver parenchyma was observed in T0 and T10, with visibly present hepatocytes, Kupffer cells, and stellate cells. In T15, there were symptoms of hepatocyte vacuolization, most likely with lipid droplets in the periportal area, but the typical parenchymal structure was still maintained. However, centrilobular inflammatory infiltration and signs of structural malformations of the parenchyma were clearly visible in T20. On the contrary, the parenchyma structure in T25 was almost normal, despite widened sinusoids.

Figure 2 illustrates central vein congestion in T0, T10, T15, and T20. Additionally, peri centrilobular lymphatic infiltration is observed in T0 and T10, albeit smaller in T15. Hepatocyte vacuolization is evident in the demonstrated photos of T10 and T25, indicating the commencement of steatosis in T25. As shown in Figure 2, in T10, the hepatic parenchyma structure is obliterated, with some pericentral necrosis present.

Referring to Figure 3, the liver parenchyma has a normal structure in T0 with some degeneration of ballooning cells. In T10, there is mild steatosis with vein congestion and periportal necrosis. T15 exhibits congestion of the central vein and sinusoids with centrilobular necrosis. Included in Figure 3, T20 shows complete disintegration of the normal parenchyma structure, probably due to tumorigenesis, with necrosis and fibrosis and signs of an inflammatory reaction. T25 displays centrilobular steatosis with lymphatic inflammation and an obliterated hepatic parenchyma structure. T15, T20, and T25 also exhibit extensive hemorrhaging, which is mostly presented in several areas near larger blood vessels (T15, T25).

### 3.2. Malondialdehyde (MDA) Concentration in the Liver of Hens

Under control conditions, a high rate of lipid peroxidation was noted at both the beginning and end of the experiment, as reflected by high MDA concentrations (Figure 4). In the experimental groups, liver MDA content depended on both the amount and the duration of lupin supplementation. As shown in Figure 4, at the 33rd day of the experiment, only the 10% inclusion of lupin in the diet showed a numerically reduced liver MDA level (*p* > 0.05) compared to the control group. Higher inclusions of lupin intensified lipid peroxidation in the liver, with significant (*p* ≤ 0.05) values at 20% inclusion. On the 66th day of measurement, lipid peroxidation was increased in the T10, T15, T20, and T25 groups compared to the control group. At 99 days, the liver MDA concentration was high in the control, T10, T20, and T25 groups. At this time, only the 15% addition of lupin to the diet significantly (*p* ≤ 0.05) reduced lipid peroxidation in comparison to the control and other experimental groups.

### 3.3. Cytosolic and Microsomal Antioxidant Capacity—Mean Activity of DT-Diaphorase, Catalase (CAT), and Ethoxycoumarin O-Deethylase in the Liver of Hens

Compared to the controls, each level of lupin supplementation inhibited diaphorase activity at day 66 of the experiment. On day 33, this inhibition was significant (*p* ≤ 0.05) only for T10 and T20 (Figure 5). On the final measurement day, only the 25% addition of lupin revealed a numerically reduced diaphorase activity, but without statistical significance. At that time, the highest (*p* ≤ 0.05) activity of this enzyme, averaging 341.32 ± 38 nmol/mg × min, was noted for 15% lupin content.

In the case of liver catalase, it was observed that, compared to the control group, each level of lupin used in the experiment significantly (*p* ≤ 0.05) reduced the enzyme activity after 66 days (Figure 6). After 33 and 99 days, no statistically significant differences in catalase activity were observed between the control group and the experimental groups. Similarly, no statistically significant differences in catalase activity were observed between the experimental groups.

After 33 and 66 days, ethoxycoumarin O-deethylase activity in the liver was highest (*p* ≤ 0.05) in birds whose diet was not supplemented with lupin (T0). Significantly lower ECCOD activity (*p* ≤ 0.001) was observed in all groups of birds fed with lupin (T10–T25) (Figure 7). However, on the 99th day of the experiment, ECCOD activity was extremely low in both the control and experimental groups, and the difference between group T0 and the other groups was slight, without statistical significance.

### 3.4. Mean Activity of Catalase (CAT) and Superoxide Dismutase (SOD) in the Serum of Hens

In groups T0, T10, and T20, statistically significant differences in the average catalase activity were observed between all sampling days. In group T0, the average catalase activity was statistically significantly higher (*p* ≤ 0.05) at the 33rd day of the study (74.69 ± 3.14) than at 66 (33.84 ± 2.42) and 99 days (28.07 ± 1.64). Similarly, the average catalase activity in group T10 was statistically significantly higher (*p* ≤ 0.05) at the 33rd day of the study (140.85 ±9.68) than at 66 (62.72 ± 9.47) and 99 (91.56 ± 3.77) days. In group T10, the average catalase activity increased statistically significantly at the 99th day of the experiment compared to its activity at 66 days. Statistically significantly higher (*p* ≤ 0.05) catalase activity in group T20 was observed at the 66th day of the study (97.89 ± 3.10) compared to the other days. The highest catalase activity in the experiment was observed at the 99th day of the study in group T15 (179.38 ± 10.53). This value was also statistically significantly higher (*p* ≤ 0.05) than the activity of this enzyme in this group at 33 and 66 days. The most balanced catalase activity was observed in group T25; however, at the 66th day of the study, the average catalase activity was statistically significantly higher (*p* ≤ 0.05) than at 33 and 99 days. At the 33rd day of the study, the average catalase activity varied greatly between groups. The average catalase activity in group T10 on that day (140.85 ± 9.68) was statistically significantly higher (*p* ≤ 0.05) than in the other groups. After 66 days, the average catalase activity in group T0 differed statistically significantly from the other groups. At the 99th day of the study, statistically significant differences in average catalase activity were observed between all groups, with the highest activity observed in group T15 (179.38 ± 10.53b) (Figure 8).

The average superoxide dismutase (SOD) activity in group T0 was statistically significantly higher (*p* ≤ 0.05) (0.19 ± 0.02) after day 33 of the study than after 66 and 99 days. In groups T10, T20, and T25, statistically significantly higher (*p* ≤ 0.05) average SOD activity was observed at day 66 of the experiment, compared to the other days (day 33 and 99). Moreover, in groups T10 and T25, SOD activity levels at days 33 and 99 did not differ statistically significantly. However, the average SOD activity in group T20 after 99 days (0.06 ±0.03) was statistically significantly lower (*p* ≤ 0.05) than on the other days of the study. The lowest average SOD activity at day 33 of the study was observed in group T15, and was statistically significantly higher (*p* ≤ 0.05) than on the remaining days (66 and 99). At day 33 of the study, SOD activity was highest in group T0 (0.19 ± 0.02), and this value was statistically significantly different from that observed in the other experimental groups. SOD activity at 33 days was statistically significantly lower (*p* ≤ 0.05) in group T15 than in groups T10 and T20. At 66 days, the average SOD activity was statistically significantly higher (*p* ≤ 0.05) in groups T10 and T20 than in groups T15, T25, and T0. After 99 days of the experiment, the average SOD activity in groups T15 and T20 differed statistically significantly from the activity in group T25, in which the highest SOD activity was recorded (0.13 ± 0.02), (Figure 9).

Using the *t*-test for independent groups, the activities of superoxide dismutase and catalase were compared in the T00 reference group (24 weeks of age) with the remaining experimental and control groups (day 33 of experiment, i.e., 29 weeks of age), respectively. SOD activity was significantly higher in the T00 group compared to the T10 (0.14 ± 0.018; *p* < 0.001), T15 (0.1 ± 0.015; *p* < 0.0001), T20 (0.14 ± 0.023; *p* < 0.0001), and T25 (0.12 ± 0.02; *p* < 0.0001) groups, and did not differ statistically significantly for the T0 group (0.18 ± 0.021; *p* = 0.20) (Table S1). In the case of catalase, the enzyme activity was significantly lower (*p* ≤ 0.05) in the T00 group (39.93 ± 5.85) compared to the other groups (*p* < 0.0001 for the T0 (74.68 ± 3.44), T10 (140.84 ± 10.59), T20 (57.58 ± 3.87), and T25 (58.89 ± 2.09) groups, and *p* < 0.05 for in the T15 group (46.07 ± 2.39)) (Table S1).

## 4. Discussion

The effects of ROS result in the intensification of lipid peroxidation [46], which leads to the breakdown of membrane polyunsaturated fatty acids and modification of the structure of cell membranes [47]. Previously published results indicate that the long-term use of Lupinus angustifolius L. protein hydrolysate in the diet of mice reduces the concentration of lipid peroxidation products in the liver, including malondialdehyde (MDA), thus preventing MAFLD (metabolic-associated fatty liver disease) [48]. Similarly, the use of Lupinus albus seed extract in mice with type 1 diabetes reduced MDA concentration in the muscle, liver, kidney, and brain tissues, inhibiting lipid peroxidation and ensuring antioxidant protection [49]. Although MDA is a well-established marker used to investigate the oxidative damage of lipids, it should be undertaken that the formation of MDA, and the scale and rate of lipid oxidation in the tissues of living organisms, is influenced by a number of endo- and exogeneous factors, such as availability of substrates, age, and body weight [50]. It should also be assumed that the intensive productivity of laying hens, accompanied by intense liver metabolism, may result in a variation in hepatic MDA concentration. In the present study, the use of high levels of lupin, especially 20%, in the diet of laying hens caused an increase in MDA concentration in the liver after just 33 days of use. This was observed concomitantly with DT-diaphoraze inhibition. Slightly elevated MDA concentration was also noted after 99 days in layers whose diet was supplemented with 10% and 25% lupin. This observation was also noticed together with the depletion of liver antioxidant capacity. Both significant and insignificant increases in MDA concentration on these days indicate damage to liver cells by ROS and the development of inflammation. This hypothesis is supported by research in a human model of non-alcoholic steatohepatitis (NASH), in which an increase in the MDA concentration in the serum and tissues accompanied steatosis and was one of the markers of the development of NAFLD [51]. Similarly, in some cases in our study, the increase in the concentration of MDA in the liver was coexistent with lipid vacuolization of the hepatocytes after just 33 days of supplementation with lupin, especially at 15% and 20%. Furthermore, centrilobular inflammatory infiltration was clearly visible in livers exposed to 20% lupin supplementation, which was revealed on representative slides. The literature data indicate that lupin seeds contain biologically active substances with high antioxidant potential [52,53], suggesting that they can be used as nutraceuticals in the prevention and treatment of various diseases. In the present study, however, the analysis of MDA concentrations in the case of the long-term use of lupin in laying hens did not show this potential. Increased lipid peroxidation associated with oxidative damage, as evidenced by an increase in liver MDA concentration in the T20 and T25 groups, lead to progressive hepatic steatosis in laying hens. These changes indicate a pathological process that ultimately ends in liver failure and, after a long period of use of lupin, may increase the mortality of laying hens. These observations were confirmed not only by the presence of numerous foci of steatosis, but also by progressive fibrosis and necrosis of the liver visible in the histopathological samples, especially after 66 and 99 days of lupin supplementation. According to current knowledge [54], steatosis and fibrosis of the liver in humans is a predisposing factor for the development of hepatocellular carcinoma (HCC). In laying hens, of course, the development of liver cancer is of little importance, especially in terms of productivity. Nevertheless, given the role of fibrosis and steatosis in hepatocarcinogenesis, the study showed that in laying hens receiving lupin at 20% for 99 days, a total breakdown of the structure of the liver parenchyma was observed, most likely due to neoplasm, with visible necrotic lesions. It should also be noted that these changes are accompanied by numerous infiltrations of inflammatory cells, which by performing their functions are an additional source of oxygen free radicals, released during respiratory burst. Taking into account our results, we can suppose that the supplementation of poultry diets with lupin seeds, especially at high levels, may contribute to disturbances of metabolic processes in the liver and also negatively affect the balanced immune response, stress response, and free radical production, up to and including hepatocarcinogenesis. However, these observations require further analysis, especially in terms of the histopathological evaluation of the liver combined with the analysis of immunological and functional parameters of this organ. DT-Diaphorase (DTD) is a flavoprotein which exists as a dimer, of which each unit has a molecular weight of 32,000 kD and is associated with a flavin adenine dinucleotide (FAD) group, noncovalently attached to the protein [55]. DTD is predominantly a cytosolic enzyme (over 90%), but is also found in the endoplasmic reticulum, mitochondria, Golgi body, and nucleus. According to current findings, DTD may exert both pro-and antioxidant effects [56]. However, as oxidative stress is characterized by quinone cytotoxicity, and DT-diaphorase as a flavoenzyme is actively involved in quinone detoxification, the characteristics of an antioxidant enzyme are ascribed to it. The antioxidant function of DTD is also associated with the recycling of the membrane antioxidants ubiquinone and vitamin E [55]. Moreover, DTD acts as a Phase II detoxification enzyme, with the detoxifying step bypassing the formation of free radicals, thus protecting tissues against mutagens, carcinogens, and cytotoxics [57]. While DTD has a high level of activity in human extrahepatic tissues, in other mammals, such as rats, monkeys, and dogs, this enzyme is most active in the liver [58]. However, its activity in the liver of poultry, including laying hens, is not fully known. In the present study, in laying hens that were not exposed to the effects of lupin, the factor changing this activity was the age of the birds. However, this was not a linear relationship, as the maximum activity of the enzyme was observed at day 66 of the experiment. DTD activity decreased on the 66th day of the study in all experimental groups, which may indicate that lupin does not have an antioxidant effect. On the other hand, it was on the last day of the experiment (99) that a significant increase in DTD activity was noted in hens receiving 15% lupin. It is worth noting that in the same experimental conditions, necrotic foci might develop in the liver of laying hens, together with the infiltration of inflammatory cells. Although at that time DTD activity declined under the influence of 20% lupin, not only the necrosis and fibrosis of liver tissue, but also neoplastic changes in selected liver samples were observed. According to recent studies [55,58], the overexpression of DTD accompanies neoplastic changes in the liver, with no change in activity in the surrounding healthy tissue. With this in mind, the use of DTD is postulated in targeted anticancer treatment. Since DTD acts to detoxify and protect the cell from toxins and mutagens, the upregulation of DTD may be part of the early carcinogenic process and can be treated as an early marker of hepatocarcinogenesis [59]. This observation may be relevant to our study, in which the long-term use of high levels of lupin in the diet of laying hens destroyed cell structures (hepatocytes), which can be observed in the histopathological photos presented in the work, leading to liver failure, which later may negatively affect the overall health status of hens and reduce production rates.

An imbalance between the production and removal of free radicals results in oxidative stress, causing the body to activate natural mechanisms aimed at restoring the redox balance, in part by eliminating free radicals and their derivatives [31,60,61]. This is achieved through the activation of numerous antioxidant enzymes, among which an important role is played by superoxide dismutase (SOD), which converts superoxide anion to hydrogen peroxide, and by catalase, which catalyzes the conversion of hydrogen peroxide to water and molecular oxygen [31,60,61]. The present study showed that for each level of lupin used in the experiment (groups T10–T25), serum SOD activity at 33 days was lower than in the control group. These results indicate that the inclusion of lupin in the diet for a short time does not affect the activity of antioxidant enzymes, and thus does not affect the oxidant/antioxidant balance in laying hens. A statistically higher activity of this enzyme was noted on the 66th day of the experiment in the T10, T20, and T25 groups, and on the 99th day in the group receiving 25% lupin (T25). This may indicate that the long-term use of lupin in laying hens reduces oxidative stress by increasing SOD activity. Santos-Sánchez et al. [62] demonstrated that the administration of lupin (*L. angustifolius*) protein hydrolysate (LPH) to mice increases the enzymatic activity of SOD and CAT, as well as TAC (total antioxidant capacity) in the plasma, and Guo et al. [63] additionally showed that LPH increases the enzymatic activity of SOD and GPx (glutathione peroxidase) in the HepG2 line of human hepatocytes. These studies show that protein hydrolysates derived from lupin exert a protective effect by reducing oxidative stress. Natural antioxidant systems based on the activity of specific enzymes, additionally supported by the dietary stimulation of their production, can lead to an increase in SOD activity and antioxidant defense, or to a decrease in SOD activity and the activation of other antioxidant mechanisms [61]. In our experiment, we used whole lupin seeds, which contain a variety of biologically active substances that presumably might also affect non-enzymatic antioxidant defense mechanisms. It should be stressed that SOD activity usually decreases in conditions of severe stress, and the antioxidant system becomes ineffective due to the overproduction of ROS. Therefore, it is likely that the low SOD activity in the serum at 99 days of the experiment in experimental groups T10–T20 was due to this effect of lupin, i.e., its inclusion in the diet caused severe stress in laying hens during the period of high production. However, as mentioned previously, increasing the dose of lupin from 20 to 25% in the feed for long-term use (99 days) may improve serum antioxidant capacity.

Human studies on catalase (CAT) have shown that its activity in the plasma increases in the case of diseases accompanied by inflammation, due to its release from damaged cells (mainly erythrocytes) [64,65]. In addition, it has been demonstrated that an increase in the activity of this enzyme during inflammation may be treated as a marker of the body’s defense response [66]. In the present study, in all experimental groups of laying hens receiving various amounts of lupin in their diet, beginning at 66 days of the experiment there was an increase in serum catalase activity in comparison to the control group. In addition, the higher the level of lupin added to the feed, the more severe steatosis was observed in the histopathological examination, as was depicted in the chosen slides. On the one hand, it should be emphasized that the increase in catalase activity in serum was most likely associated with inflammatory processes in the liver and an increase in ROS concentration, which could lead to disturbances in redox processes due to damage to hepatocytes. On the other hand, diets containing a high percentage of fats increase fatty acid oxidation by activating the peroxisomal oxidation pathway associated with the overproduction of H_2_O_2_. However, an increase in CAT activity would suggest a compensatory effect [67,68]. Such observations may relate to our results, where the % raw fat content in the feed with lupin reached a value of up to 10.52% in the T25 group compared to the control group (T0), where this value was 8.50%. A higher fat content in the feed results in an increase in the concentration of catalase in the serum, which provides protection against the increasing processes of fatty acid oxidation and H_2_O_2_ production. Similar observations were made by Rindler et al. [69], who showed that this enzyme is crucial in preventing oxidative damage in mice fed a high-fat diet. It is interesting that, in our experiment, a different profile of this enzyme was observed in the liver. Beginning on day 66 of the experiment, low activity or the absence of changes in catalase activity in the liver cells was noted in comparison to the control. Similar results were obtained in a study in mice, in which steatosis associated with the development of non-alcoholic steatohepatitis (NASH) and non-alcoholic fatty liver disease (NAFLD) was accompanied by a reduction in the activity of antioxidant enzymes, including catalase, in hepatocytes [70]. These results may support the hypothesis that excessive oxidative stress induced by inflammatory processes leading to steatosis, as well as excessive ROS production, can inactivate and/or reduce catalase activity. Similar observations were made by Noeman et al. [71], who showed increased oxidative stress in liver, heart, and kidney tissues in rats with obesity induced by a high-fat diet, characterized by no changes or a reduced activity of antioxidant enzymes, including CAT. Although the results of research on catalase activity in steatosis in humans are often conflicting, the overproduction of ROS and disease progression are undoubtedly conducive to the inactivation of antioxidant enzymes, disturbing the oxidant/antioxidant balance [72,73]. The literature data together with the results of the present study suggest that high levels of lupin in the diet may induce steatosis of the liver, thereby increasing ROS production and possibly reducing the synthesis of antioxidant enzymes in the organs, disturbing homeostasis in the body.

Cytochrome P450 (CYP) is a complex and very large superfamily of enzymes which play a major role in the metabolism of xenobiotics, as well as in the oxidation of endogenous compounds [74]. Exposure to certain drugs or other substances can induce the synthesis of some CYP enzymes, which accelerates the metabolism of other drugs that are substrates of these enzymes. On the other hand, certain substances can inhibit the specific P450 isoenzyme-mediated metabolism of other drugs in a dose-dependent fashion. These substances include natural flavonoids [75]. The literature indicates that the activity of hepatic CYP-dependent 7-ethoxycoumarin deethylase (ECOD) decreases in rats which have received flavonoids [75]. Lupin seeds are known to be rich in polyphenols, phytosterols, and squalene [12,19]. The most abundant phenolic compounds in L. angustifolius seeds are flavones, phenolic acids, and isoflavones, which represent 76%, 19%, and 4% of the total identified phenols, respectively [28]. Although many authors suggest that lupin is a good and inexpensive source of protein in the diet, which translates to health and production benefits in animals, our study confirms that as in other species, the flavonoids contained in lupin can significantly inhibit ECOD activity in the hepatic microsomes of laying hens. Irrespective of the level of inclusion of lupin and the duration of supplementation in our study, the activity of this enzyme was extremely low. It should be emphasized that except for day 99 of the experiment, microsomal ECOD activity remained high in laying hens that did not receive lupin in their diet. We can assume that the decreasing ECOD activity on the last day of experiment could have been related to the physiological depletion of CYP enzymatic activity. This seems to be possible if we take into account the higher metabolic liver activity of laying hens, and also the fact that the 99th day was the peak time of productivity of these animals. Given the involvement of CYP enzymes, including 7-ethoxycoumarin deethylase, in the biotransformation of endogenous and exogenous chemical compounds, a reduction in enzyme activity will be associated with a decrease in the detoxifying capacity of the liver. This further entails metabolic disorders in the liver and a reduction in immunity, especially in birds exposed to multiple stress factors, as in the case of laying hens, e.g., during periods of increased laying.

It is worth emphasizing that the period of intensive laying and the age of layers may themselves induce oxidative stress, which causes an increase in the activity of antioxidant enzymes. This is evidenced by the statistically significantly higher CAT activity at 33 days in the control group and experimental groups in comparison to its activity before the start of the experiment, i.e., when the birds were 24 weeks old (Table S1). The age of hens is also a significant factor increasing lipid peroxidation and protein oxidation, which lead to the delayed production and recovery of antioxidants, resulting in disturbances to liver regeneration processes [76,77]. Increased lipid deposition in the liver, associated with the duration of the experiment and supplementation with high-protein lupin, which stimulated laying [12], may therefore be regarded as an explanation for the reduced antioxidant capacity of the liver in laying hens.

## 5. Conclusions

To sum up, it should be stated that high doses of lupin may cause fatty liver processes and reduce the synthesis of antioxidant enzymes in the organs, contributing to the disturbance of homeostasis in the body. In some circumstances, an increase in MDA concentration in the liver indicates cell damage as a result of increased lipid peroxidation by ROS and the development of inflammatory processes. Damage to liver cells in laying hens is additionally manifested by the presence of steatosis and the progressive processes of fibrosis and necrosis of this organ, especially after 66 and 99 days of lupin use. Low activity of the 7-ethoxycoumarin deethylase enzyme additionally indicates a reduction in the detoxification capacity of the liver, leading to metabolic disorders. Including lupin in the diet for a short time does not affect the activity of antioxidant enzymes and, therefore, does not affect the oxidation–antioxidant balance in laying hens. Further research is necessary on the mechanisms initiating changes at the cellular, subcellular, and molecular levels activated by the bioactive compounds contained in lupin.

## Figures and Tables

**Table 1:** Composition of control and experimental diets.

Components, %	Group				
	T0	T10	T15	T20	T30
Wheat	57.78	53.324	50.737	48.153	45.568
Post-extraction soybean meal	23.71	17.678	14.727	11.776	8.825
*Lupinus angustifolius*	-	10	15	20	25
Calcium carbonate	10.219	9.812	9.793	9.773	9.754
Rapeseed oil	5.976	6.77	7.268	7.766	8.264
Monocalcium phosphate	0.99	1.002	1.009	1.015	1.022
Premix without coccidiostat ^1^	0.5	0.5	0.5	0.5	0.5
NaHCO_3_	0.294	0.312	0.32	0.329	0.337
DL-Methionine	0.234	0.251	0.261	0.27	0.279
Sodium chloride	0.171	0.156	0.15	0.143	0.136
Lysine HCL	0.07	0.11	0.129	0.148	0.167
Threonine	0.029	0.038	0.042	0.047	0.051
L-Valine	0.017	0.033	0.042	0.05	0.059
Optiphose (0.01%) ^2^	0.01	0.01	0.01	0.01	0.01
l-Tryptophan	-	0.004	0.012	0.02	0.028

^1^ Provided per kg diet: vit. A—10,000 IU; vit. D3—2000 IU; vit. E—20 mg; vit. K3—1.5 mg; vit. B1—1 mg; vit. B2—4 mg; vit. B3—20 mg; vit. B5—8 mg; vit. B6—1.5 mg; vit. B9—0.8 mg; choline—200 mg; Fe—45 mg; Mn—90 mg; Cu—8 mg; Zn—60 mg; I—1 mg; Co—0.5 mg; Se—0.25 mg; antioxidant—15 mg; vit. B12—3300 mcg; biotin—50 mg. ^2^ Optiphose—6-phytase derived from *E. coli.*

**Table 2:** Nutritional value of control and experimental diets.

Nutritional Value of the Mixture ^a^, %	Group				
	T0	T10	T15	T20	T25
AMEn ^1^	2850	2850	2850	2850	2850
Total protein	17.99	17.89	17.88	17.92	17.95
Raw fat	8.50	8.48	9.16	9.84	10.52
Raw fiber	3	3.99	4.48	4.97	5.47
Digestible lysine	0.84	0.84	0.84	0.84	0.84
Digestible threonine	0.59	0.59	0.59	0.59	0.59
Valine	0.7	0.7	0.7	0.7	0.7
Methionine + cystine	0.76	0.76	0.76	0.76	0.76
Available phosphorus	0.5	0.5	0.5	0.5	0.5
Calcium	4.29	4.29	4.29	4.29	4.29
Chlorine	0.16	0.16	0.16	0.16	0.16
Sodium	0.18	0.18	0.18	0.18	0.18

^1^ AMEn—apparent metabolizable energy corrected to zero nitrogen retention. ^a^—values analyzed.

## Data Availability

All data generated or analyzed during this study are included in this published article, and are available on request from the corresponding author.

## Supplementary Materials

The following supporting information can be downloaded at: https://www.mdpi.com/article/10.3390/antiox13020251/s1, Supplementary Table S1: Comparison of superoxide dismutase (SOD) and catalase (CAT) levels for experimental groups T00–T25 using one-way ANOVA and Tukey’s test (small letters—a) and activity of the mentioned enzymes between group T00 at 24 weeks of age and groups T0-T25 at 29 weeks of age (day 33 of the experiment)—capital letters (A, B)—in the serum of laying hens. The results are presented using means and standard deviation (±SD) for *p* ≤ 0.05. Statistical significance was indicated as follows: significant differences ** for *p* ≤ 0.001; *** for *p* ≤ 0.0001; N—number of hens.
