# Supplementary material for: Liver Antioxidant Capacity and Steatosis in Laying Hens Exposed to Various Quantities of Lupin (Lupinus angustifolius) Seeds in the Diet
Source: Antioxidants (Basel). 2024 Feb 19;13(2):251. doi: 10.3390/antiox13020251 (PMC10886069; doi:10.3390/antiox13020251)
Supplement: Supplementary file 1 [file antioxidants-13-00251-s001.zip › antioxidants-2839142-supplementary.pdf]

antioxidants

MDPI

Table S1. Comparison of superoxide dismutase (SOD) and catalase (CAT) levels for experimental groups T00—T25 using one-way ANOVA and Tukey's test (small letters – a) and activity of the mentioned enzymes between group T00 on 24 weeks of age and groups T0-T25 on 29 weeks of age (33 day of the experiment) - capital letters (A, B), in the serum of laying hens. The results are presented using means and standard deviation (±SD) for $p \le 0.05$. Statistical significance was indicated by: significant differences ** for $p \le 0.001$; *** for $p \le 0.0001$; N — number of hens.

		Serum	
		SOD [U/mL]	Catalase [µM/min/mL]
24 weeks of age (N = 30)	T00	0.2±0.02A	39.94±5.34A
33 day of experiment, i.e. 29 weeks of age (N = 30)	T0	0.19±0.02Aa***	74.69±3.14 B***a***
	T10	0.14±0.02B***a***	140.85±9.68 B***a***
	T15	0.11±0.02 B***a***	46.08±2.19 B*a***
	T20	0.15±0.03 B***a***	57.58±3.53 B***a***
	T25	0.12±0.02 B***a**	58.9±1.91 B***a**
